# Biological Activity of *Bursera schlechtendalii* Essential oil and the Roles of Its Chemical Components in the Wound Healing Process

**DOI:** 10.3390/ijms241311040

**Published:** 2023-07-03

**Authors:** Lesslie Espinosa-Espinosa, Octavio Canales-Alvarez, Marlene Guadalupe Rodríguez-López, César Antonio Flores-Tinajero, Maria Margarita Canales-Martinez, Marco Aurelio Rodriguez-Monroy

**Affiliations:** 1 Laboratorio de Investigación Biomédica de Productos Naturales, Facultad de Estudios Superiores Iztacala, Universidad Nacional Autónoma de México, Tlalnepantla de Baz 54090, Mexico; biol.lespinosa@gmail.com (L.E.-E.); octaviocanalesa@gmail.com (O.C.-A.); 2 Laboratorio de Farmacognosia, UBIPRO Facultad de Estudios Superiores Iztacala Universidad Nacional Autónoma de México, Tlalnepantla de Baz 54090, Mexico; rodriguez.lopez.marlene.g@gmail.com (M.G.R.-L.); dra.margaritacanales@gmail.com (M.M.C.-M.); 3 Laboratorio de Fitoquímica II, Escuela Nacional de Ciencias Biológicas, Instituto Politécnico Nacional, Mexico City 07738, Mexico; cflores@ipn.mx

**Keywords:** wound healing, essential oil, *Bursera*, monoterpenes

## Abstract

Essential oils are composed of terpenes, some of which have properties related to healing. *Bursera schlechtendalii* essential oil (BSEO) is used to heal superficial wounds. However, there have been no studies verifying this property. The objectives of this study were to evaluate the healing activity of BSEO in a murine model and to propose the roles of its chemical components in this process. Healing activity was evaluated by an incision model, histological analysis was performed, and tensile strength and antibacterial activity were measured. The chemical composition of BSEO was determined by gas chromatography coupled with mass spectrometry (GC–MS), and the mechanisms of action of each chemical component during the phases of the healing process were proposed. In addition, acute dermal toxicity was evaluated. BSEO showed better wound closure at the macroscopic, histological, and tensile strength levels compared to controls and had an antibacterial effect. The major compound in BSEO was α-phellandrene. However, most of the monoterpenes identified in BSEO were in agreement with information found in the literature, so the possibility of synergy between the chemical components and their different targets in the healing process was schematically proposed. BSEO was shown to be safe in the dermal toxicity evaluation.

## 1. Introduction

The Burseraceae family is distributed from the southern United States to South America, showing diversity on the Mexican Pacific slope, where more than 70 endemic species are found. Within this family, 18 genera have been classified, among which three are present in Mexico: *Beiselia, Protium* and *Bursera* [1]. The stems, leaves and fruits with sweet and strong aromas from the species in the genus *Bursera* are known to be used for synthesizing essential oils.

Essential oils are mainly composed of a group of chemicals called terpenes (approximately 90% of their composition), which are organic molecules derived from isoprene (a hydrocarbon with five carbon atoms). The biological roles of terpenes include repelling insects to avoid herbivory, attracting pollinating organisms, producing allelopathy, protecting the plant from microbial diseases, and giving the characteristic aroma and flavor of certain plant species [2]. In addition, terpenes have a wide variety of pharmacological properties that are closely related to healing, such as antimicrobial, anti-inflammatory, antioxidant, macrophage migration inhibitory factor (MIF), edema reduction and decreased leukocyte migration activities [2,3,4].

Wounds are damage to the continuity of the epithelium due to accidental or intentional factors or the consequence of a disease [5]. Depending on the depth, healing time, form, contamination, etc., wounds can be classified into different categories [6,7]. In particular, superficial wounds affect the first two layers of the skin, and although they are not considered serious injuries, in most cases, adequate care is not taken for their recovery. Therefore, the process of healing is hindered, causing consequences such as a reduction in the quality of life of patients, economic losses, and psychological damage due to long periods of pain and discomfort [7].

The species *Bursera schlechtendalii* is bush commonly known as “copalillo” in the community of San Rafael, Coxcatlán in the state of Puebla, Mexico, and the essential oil extracted from its young stems is used to relieve superficial wounds [8]. This plant is characterized by being transparent in color and having a strong aroma. Regarding its chemical composition, it is known that the main components are terpenes and hydrocarbon compounds [9,10]. However, there no studies have scientifically verified its traditional use or investigated the possible mechanisms of action of its components in the healing process. Based on the traditional use of essential oil from certain species of the genus *Bursera* for wound healing and the pharmacological properties of terpenes, critical compounds found in essential oils, *B. schlechtendalii* essential oil (BSEO) may demonstrate therapeutic activity in superficial wounds. For this reason, the aim of this work was to evaluate the healing activity of BSEO in a murine model and to propose the roles of its chemical components in the different phases of healing according to the results obtained and information found in the literature.

## 2. Results

### 2.1. Wound Healing Activity

#### 2.1.1. Macroscopic Observations and Wound Contraction Measurements

**Figure 1:**
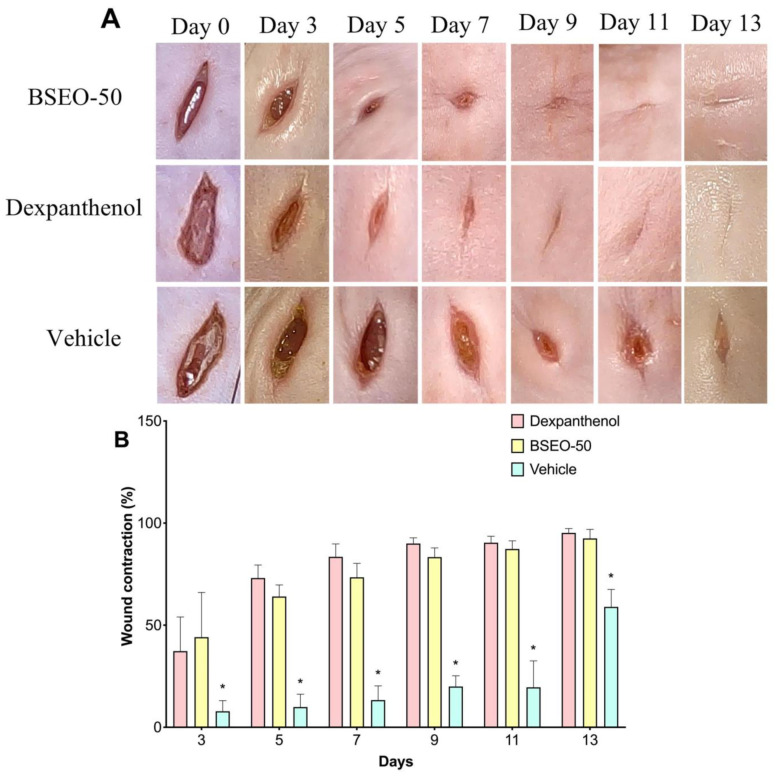
Effect of BSEO-50 on wound contraction. (**A**) Macroscopic observations of the course of wound closure from Days 0–13 in the BSEO-50 (50% *v*/*v*), control (0.5 mg of dexpanthenol) and mineral oil (vehicle) groups. (**B**) Percentages of wound contraction in the control group and BSEO-50-treated group. All values are expressed as percent wound contraction ± SEM. Two-way ANOVA was performed with Tukey’s multiple comparison analysis, and the dexpanthenol and BSEO-50 groups had similar wound closure. * *p* < 0.05 compared to the dexpanthenol and BSEO-50 groups.

Macroscopic observations of wounds treated with the reference drug (dexpanthenol) showed a more defined wound in contrast to BSEO-50-treated group. In the case of the control group, granulation tissue was observed from the 3rd day, while this type of tissue was observed on the 5th day in the BSEO group. On Day 11, the scar was defined in the dexpanthenol and BSEO-50 groups. In the group treated with mineral oil, it was not possible to observe a defined scar during the 14 experimental days (Figure 1A). The length of the wound closure did not show significant differences (*p* < 0.05) between the control group and BSEO-50 group. In addition, on the seventh day, 75% closure was achieved in both of these groups (Figure 1B).

#### 2.1.2. Histological Analysis

**Figure 2:**
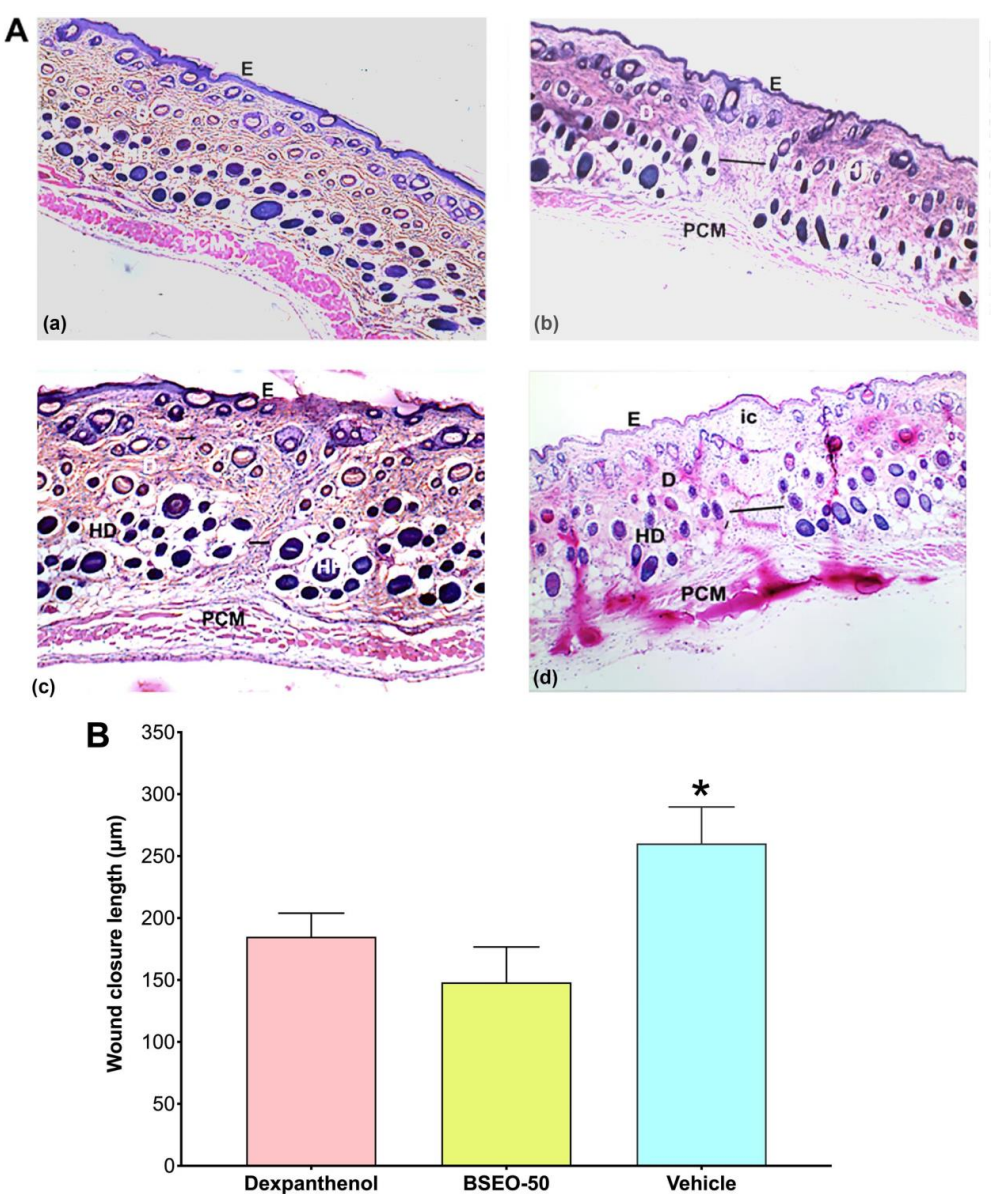
(**A**) Histological analysis after BSEO-50 treatment. (**A**) Histological sections of mouse skin wounds. All photos were taken at 10× magnification. The group treated with 0.5 mg of dexpanthenol showed a thicker epidermis than the healthy skin group and greater cellular infiltrate than the BSEO-50 (50% *v*/*v*) group. Representative photographs of wound architecture on Day 14. Normal skin (**a**), dexpanthenol (**b**), BSEO-50 group (**c**), and mineral oil (vehicle) (**d**). Epidermis (E), dermis (D), hypodermis (HD), hair follicle (HF), panniculus carnosus muscle (PCM), inflammatory cell (ic), wound measurement (black lines), and collagen fibers (←). The tissue was stained with H&E and visualized at 10× magnification. (**B**) Wound reduction on Day 14 of treatment. The results are expressed as the mean ± S.D. Data analysis was performed by one-way ANOVA with Tukey’s multiple comparison post hoc test. * *p* < 0.05 compared to dexpanthenol and BSEO-50 group.

Tissue sections obtained on Day 15 were stained with the Hematoxylin and Eosin (H&E) technique to determine the architecture of the skin layers, cell infiltration, closed wound length, and collagen fiber deposition (Figure 2A). The architecture of the three skin layers was distinguished, as was hair follicles and panniculus carnosus muscle in the normal skin group (2Aa). The dexpanthenol group showed a thicker dermis than the healthy skin group and presented edema in the lesion zone (2Ab). In BSEO-50 group, collagen fibers were identified; however, increased edema formation was also observed (2Ac). However, the sizes of the wounds were not significantly different between the dexpanthenol and BSEO-50 groups *(p* < 0.05). Additionally, the group treated with the vehicle showed larger wound lengths and abundant cellular infiltrate in the epidermal layer (Figure 2Ad). Therefore, the vehicle group presented significant differences with respect to the group treated with BSEO-50 (Figure 2B).

#### 2.1.3. Tensiometric Method

**Figure 3:**
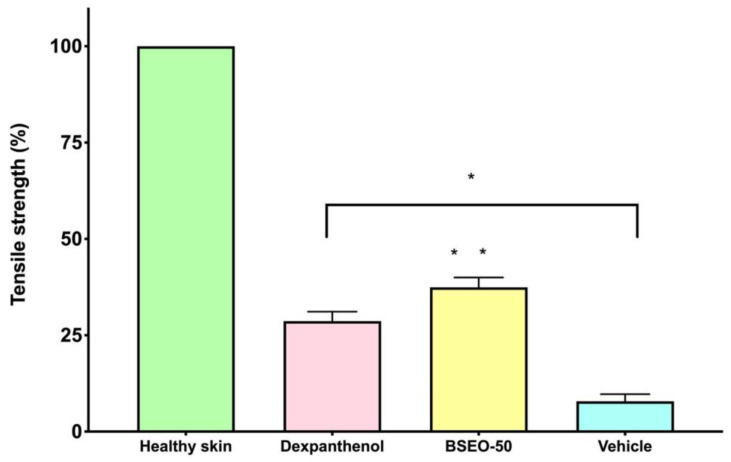
Tensile strength of BSEO-50. * The dexpanthenol (0.5 mg), BSEO-50 (50% *v*/*v*)*,* and mineral oil groups showed significant differences with respect to the healthy skin group (*p* < 0.05). ** Indicates significant differences with respect to the dexpanthenol and mineral oil groups (*p* < 0.05).

Dexpanthenol, BSEO-50 and mineral oil groups showed significant differences with respect to the healthy skin group (Figure 3). BSEO-50 also showed statistically significant differences compared with the dexpanthenol (28.68%) and mineral oil (7.88%) groups with a tensile strength percentage of 37.44% (Figure 3).

### 2.2. Anti-Inflammatory Activity in the 13-acetate-12-O-tetradecanoylphorbol (TPA)-Induced Ear Edema Model

**Figure 4:**
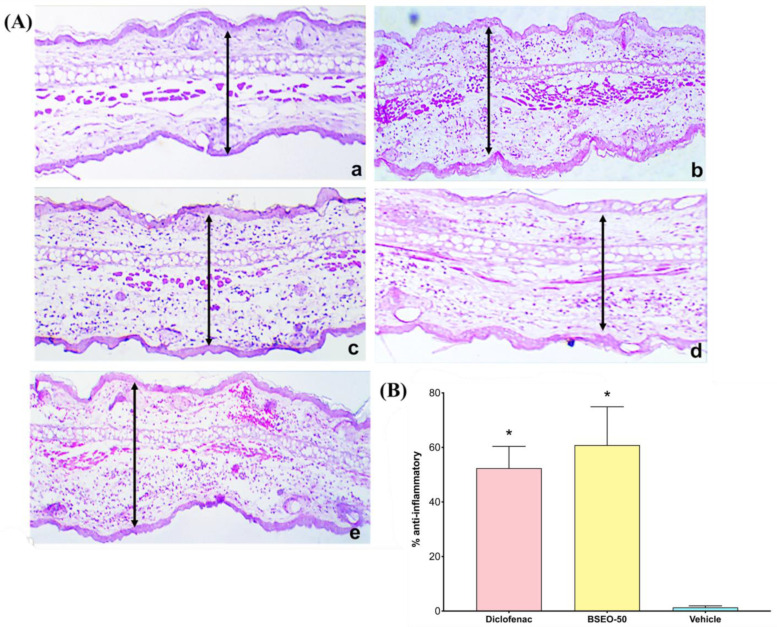
(**A**) Histological sections of auricular edema after BSEO-50 treatment (10×). (**a**) Control group (left ear) showing normal pinna. (**b**) In the group treated with only TPA, abundant infiltration of inflammatory cells and edema are observed. (**c**) In the positive control group (0.116 mg diclofenac), moderate infiltration of leukocytes and edema is shown. (**d**) The group treated with BSEO-50 (50% *v*/*v*) shows little leukocyte infiltration and edema. (**e**) The group treated with mineral oil (vehicle) shows abundant leukocyte infiltration. The samples were stained with hematoxylin-eosin, and the arrows indicate the space between the epidermis and the elastic cartilage. (**B**) Anti-inflammatory effects in the TPA model expressed as a percentage. There were no significant differences between the BSEO-50 and diclofenac groups. Arrows exemplify a measurement of ear thickness The results are expressed as the mean ± S.D.M. Data analysis was performed by Wilcoxon’s *t* test with a threshold of significance of * *p* < 0.05 compared to the vehicle group.

The histological sections from the control group showed a normal architecture (Figure 4Aa), while the sections from the group treated with TPA only presented pronounced vasodilation, edema, and leukocyte infiltration (Figure 4Ab). However, the group treated with diclofenac produced abundant infiltration of inflammatory cells and edema (Figure 4Ac). Although the BSEO-50 group also presented with edema, inflammatory cell infiltration was scarce (Figure 4Ad). The histological section treated with BSEO-50 was thinner than that of the group treated with TPA. BSEO-50 did not show a significant difference (*p* < 0.05) with respect to the diclofenac group (Figure 4B). The vehicle group presented abundant cellular infiltrate in the pinna, with significant differences with respect to BSEO-50 and diclofenac groups.

### 2.3. Antibacterial Activity

In the Kirby–Baüer test, BSEO showed a minimal effect on two bacterial strains (*Staphylococcus aureus* 29213 and *Escherichia coli* 53228). However, *Staphylococcus epidermidis* and *Pseudomona aeruginosa* did not show sensitivity to the oil. BSEO was also no better than chloramphenicol. In the broth dilution test, the MIC and MBC of BSEO against *E. coli* were found to be 0.25 and 0.5 mg/mL, respectively. However, for *S. aureus*, *S epidermidis* and *P. aeruginosa,* more than 10 mg/mL BSEO is needed to determine its bactericidal and bacteriostatic concentrations (Table 1).

### 2.4. Chemical Analysis by GC–MS

**Figure 5:**
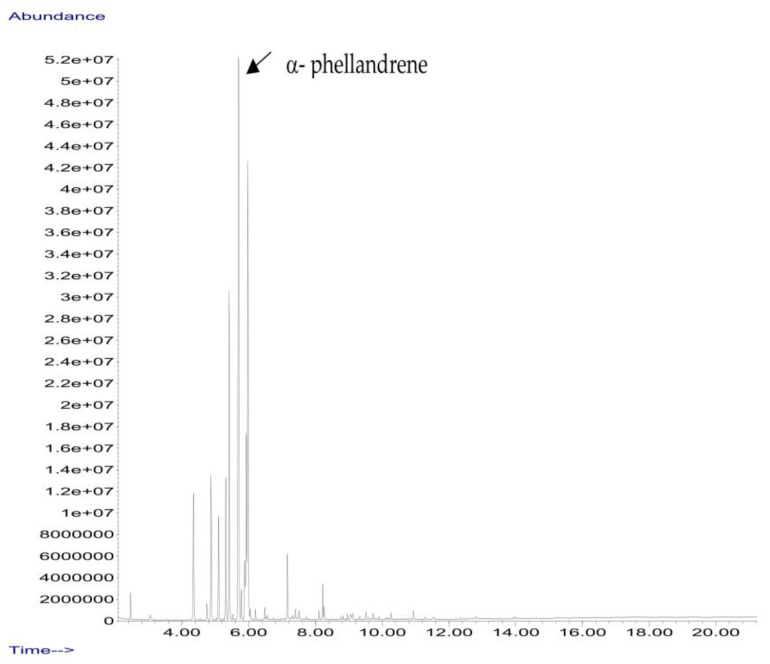
Chromatogram from *B. schlechtendalii* essential oil, highlighting the most abundant compound α-phellandrene.

*B. schlechtendalii* essential oil (BSEO, 0.5 µL) was injected (without dilution) into the GC–MS instrument, and nine compounds were identified, corresponding to 83.971% of the components belonging to the terpene family (Table 2). The most abundant compound was α-phellandrene, which was found at retention times of 5.694 min and 5.782 min and represented 31.181% of the total sample (Figure 5).

### 2.5. Proposals and Descriptions of the Mechanisms of Action of BSEO in the Healing Process

Eight of the nine chemical components that were identified in BSEO have been reported to have activities that are related to factors that could affect each of the phases in the wound healing process (Table 3), such as anti-inflammatory and antibacterial activity, a reduction in NO and ROS levels, and fibroblast proliferation and collagen production effects. This investigation considered the evaluations of pure compounds or mixtures that were tested in in vitro and in animal models.

**Figure 6:**
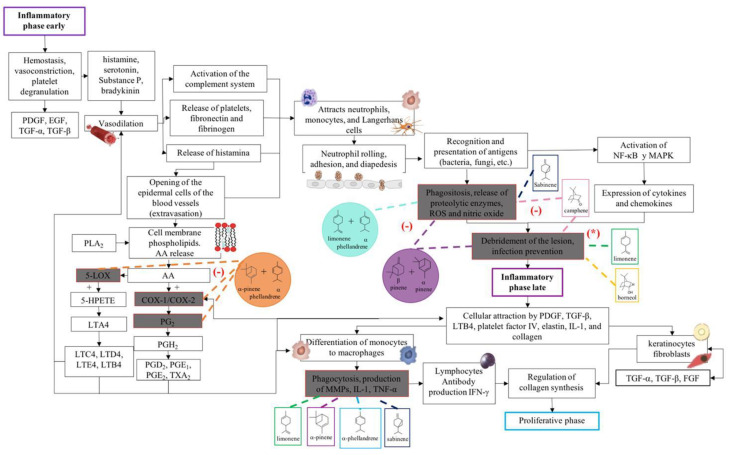
Proposed mechanisms of action of the terpenes present in BSEO in the inflammatory phase. Phospholipase A2 (PLA2), arachidonic acid (AA), cyclooxygenase-1 (COX-1), cyclooxygenase-2 (COX-2), prostaglandin G2 (PG2), prostaglandin H2 (PGH2), prostaglandin D2 (PGD2), prostaglandin E1 (PGE1), thromboxane A2 (TXA2), 5-lipoxygenase (5-LOX), 5-hydroperoxyeicosatetraenoic acid (5-HPETE), leukotriene A4 (LTA4), leukotriene C4 (LTC4), leukotriene D4 (LTD4), leukotriene E4 (LTE4), leukotriene B4 (LTB4), nuclear factor kappa, light chain enhancer of activated B cells (NF-κB), mitogen-activated protein kinase (MAPK), reactive species (ROS), platelet-derived growth factor (PDGF), transforming growth factor beta (TGF-β), metalloproteinases (MMPs), interleukin 1 (IL-1), tumor necrosis factor (TNF-α), interferon-γ (IFN-γ), d, transforming growth factor alpha (TGF-α), and fibroblast growth factor (FGF). Dotted lines indicate specific sites where monoterpenes act in the inflammatory phase of wound healing. (-) Indicates inhibition of the signaling pathway; (*) indicates antibacterial activity.

**Figure 7:**
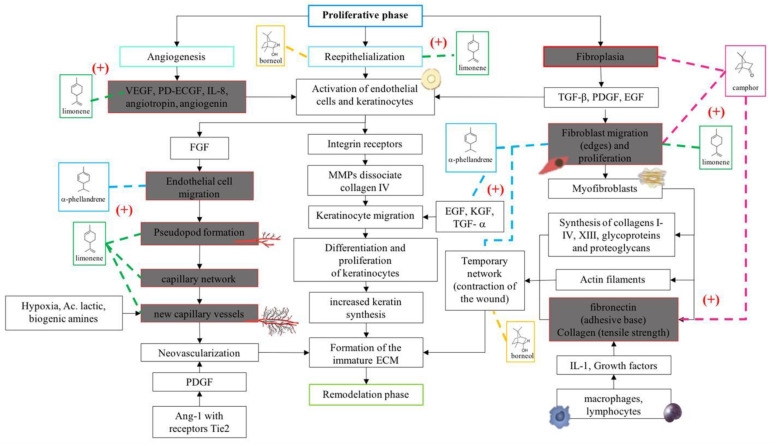
Proposals of the action mechanisms of the terpenes present in BSEO in the proliferative phase. Vascular endothelial growth factor (VEGF), platelet-derived endothelial cell growth factor (PD-ECGF), interleukin-8 (IL-8), fibroblast growth factor (FGF), metalloproteinases (MMPs), epidermal growth factor (EGF), keratinocyte growth factor (KGF), transforming growth factor alpha (TGF-α), transforming growth factor beta (TGF-β), and platelet-derived growth factor (PDGF). Dotted lines indicate specific sites where monoterpenes act. (+) Indicates activation of the signaling pathway.

**Figure 8:**
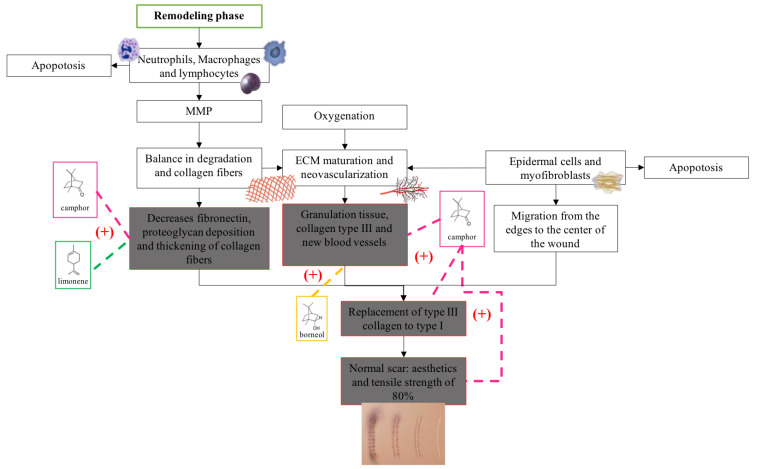
Proposals of the action mechanisms of the terpenes present in BSEO in the remodeling phase. Metalloproteases (MMPs) and extracellular matrix (ECM). Dotted lines indicate specific sites where monoterpenes act. (+) Indicates activation of the signaling pathway.

In accordance with the literature and the results obtained from BSEO, the possible mechanisms of action and the targets with which the chemical components of BSEO could act on during the inflammatory, proliferative, and remodeling phases of wound healing were schematically described (Figure 6, Figure 7 and Figure 8).

### 2.6. Acute Dermal Toxicity Test

**Figure 9:**
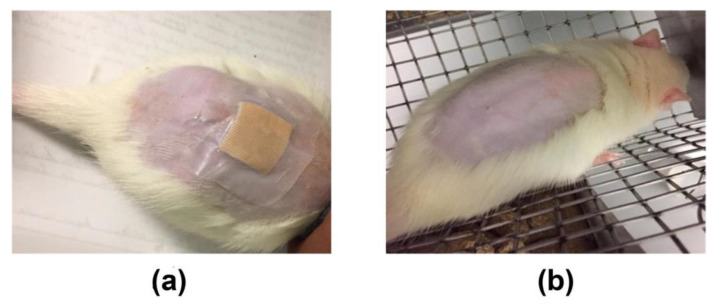
Acute dermal toxicity study. (**a**) Photographs of the contact skin at the moment of 2000 mg/kg BSEO application. (**b**) Photographs of the skin with the same dosage of BSEO on the day of sacrifice (15th day).

**Figure 10:**
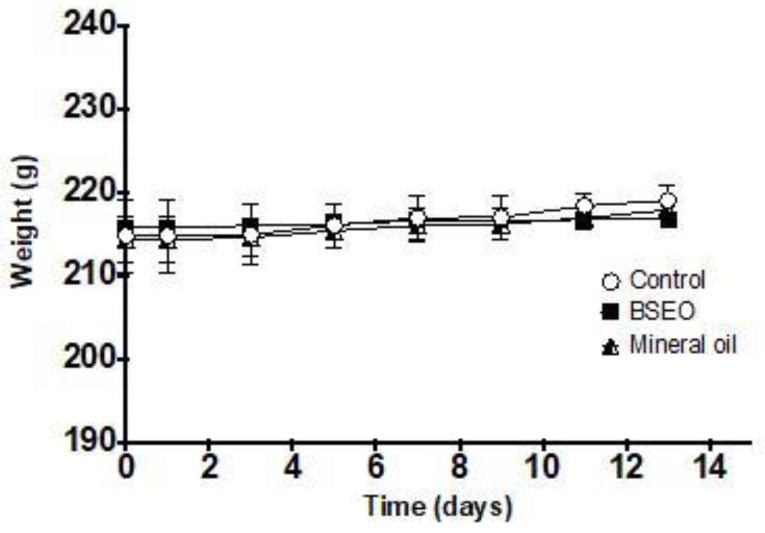
Effect of BSEO on the weights of female rats. All values are expressed as the mean ± SD (*p* < 0.05).

A BSEO dose of 2000 mg/kg was considered safe, as 24 h after application and for 14 days after the treatment, no damage to the skin or major organs was observed (Figure 9). The weights of the animals evaluated did not show significant differences during the time of exposure to BSEO or vehicles (Figure 10), changes in welfare parameters, or mortality during the 14 days after the application of the treatments (Table 4).

## 3. Discussion

Essential oils are a complex mixture of volatile, heat-labile, and aromatic compounds. They are generated in small quantities since they constitute 0.5 to 6% of the total metabolites present in a plant [20]. Terpenes are the main chemical components in essential oils, although trace amounts of phenolic compounds, alcohols, coumarins, acids, and esters can also be found [2]. The chemical composition of essential oils strictly depends on the development of the plant and the physiological response that may result after abiotic and biotic stress, which determines the quality, quantity, yield, and specific biological properties [21]. Anti-inflammatory, antibacterial, anticoagulant, and analgesic properties and effects on the migration and proliferation of endothelial cells and fibroblasts are essential for the healing process to take place, [2,22] and some metabolites present in essential oils have been shown to have one or more of these effects in vivo and in vitro.

Regarding healing efficacy, the formation of granulation tissue, the appearance of a scar, and the time required for the wound edges to close completely were evaluated macroscopically in this study. The dexpanthenol-treated group exhibited granulation tissue formation by day three, while BSEO-50 group took up to day five and the vehicle group began to show granulation tissue by day seven. In terms of edge contraction, the dexpanthenol- and BSEO-50-treated wounds displayed greater than 50% closure from Day 5, while the mineral oil group needed through Day 13. Statistical analysis of wound contraction in the dexpanthenol and BSEO-50 groups determined that there were no significant differences between them, but there were differences compared with the vehicle group. The scars that formed in the groups treated with BSEO-50 and dexpanthenol were more aesthetic and finer compared to the vehicle-treated scars. Despite this, by Day 13, all groups showed superficially closed wounds. In one study, it was observed that contraction of the wound, re-epithelialization, angiogenesis, and granulation tissue thickness were greater from the sixth day in the groups treated with limonene compared to the control groups [16].

However, histological analysis revealed that the deeper layers of the wounds were still in the process of healing. Although the positive control groups, BSEO-50 group, and the vehicle group had a thick and well-formed dermis (compared to the healthy skin group), the dermis layer showed edema formation and cellular infiltration. In particular, although BSEO-50 group presented a greater amount of edema, it also showed a greater collagen fiber formation and a better architecture of the epidermal layers (similar to that of the healthy skin group) than the dexpanthenol group. However, there was no significant difference between these groups, but there were significant differences with the vehicle group.

Tensile strength is another fundamental parameter that must be measured after a wound is generated because in normal tissue (in uninjured skin), collagen is quite strong and ordered (type I), but when an injury occurs, the collagen fibers that form are randomly arranged and smaller, providing weak tensile strength (type III). Over time, this collagen matures and forms a strong scar. However, the strength of the skin never recovers to that of what it was before injury [5,23]. The three experimental groups (BSEO-50, dexpanthenol, and vehicle) were significantly different from the healthy skin group. On the other hand, the group treated with BSEO-50 showed significant differences from the dexpanthenol (28.68%) and mineral oil (7.88%) groups, exhibiting a traction percentage of 37.44%.

Similar values have been reported in other studies, such as is the cases of the essential oils of *Lavandulae aetheroleum* (30.5%) and *Lauri aetheroleum* (27.2%), with their main components being the monoterpenes linalool and eucalyptol, respectively [24]. It should be noted that when a wound is generated, the maximum tensile strength that the recovered skin can reach is 80% [23]. In another study [25], the pure compounds α-bisabolol and α-terpineol, a sesquiterpene and a monoterpene, respectively, achieved 50% tensile strength at higher concentrations (228 and 240 μg/g mouse) than those in this study (138 μg/g mouse). Thymol (which is not found in BSEO) has been reported to promote fibroblast proliferation and collagen deposition, facilitating key events in the remodeling phase [26].

The results of the macroscopic experiment, histological analysis, and tensile strength assessments were consistent with what was found in other investigations, and this may be due to the actions of compounds present in BSEO, such as α-phellandrene, borneol, camphor, and limonene. De Christo Scherer [14], using the scratch assay in vitro model with L929 fibroblasts, concluded that α-phellandrene significantly increased the proliferation and migration of these cells compared to the control (untreated cells) in a dose-dependent manner. Borneol stimulates wound contraction, re-epithelialization, and granulation tissue formation [17]. Camphor was able to benefit skin health in a time-dependent experiment, increasing collagen III and IV levels and elastin production [19]. Limonene has also been shown to promote fibroblast proliferation and collagen fiber deposition, which was more prominent and disordered than in controls [16].

During the healing process, the inflammatory phase is crucial because the immune system activates signaling pathways to stop bleeding, activate protective cells, and prevent different factors from hindering the healing process. However, when treating a wound with an anti-inflammatory drug, several molecular pathways that can inhibit the normal course of the process are interrupted [7]. Therefore, complementary treatments that serve as modulators should be considered, reducing inflammatory signs without hindering the inflammatory phase. Therefore, this study evaluated acute inflammation by measuring the thickness of the dermal area of ear tissue through biopsies and histological techniques. BSEO-50 showed a scarce leukocyte infiltration, reduced thickness between the epidermis and the elastic cartilage (an anti-inflammatory effect), and less edema than the TPA group. Although compared to the control and diclofenac groups, BSEO-50 had displayed edema, the differences in the anti-inflammatory percentage and changes in the anatomical structure were not significant.

Carrera et al. [27] evaluated the anti-inflammatory activity of the essential oil of *B. morelensis*, both diluted by 50% with mineral oil and pure, in a plantar edema model. This activity reached percentages of 75% and 68%, respectively, which were not significantly different from the positive control (dexamethasone); the main components were α and β-phellandrene. On the other hand, diclofenac has analgesic, fever-reducing, and anti-inflammatory properties and greater pharmacological activity than other nonsteroidal anti-inflammatory drugs (NSAIDs), such as indomethacin and naproxen. Diclofenac acts on a single target during the entire inflammatory process, the COX-2 pathway, by inhibiting the production of prostaglandins [28]. Different essential oils from other plant species have been evaluated against TPA-induced inflammation and have shown which mixtures have a greater anti-inflammatory effect than the pure compounds [2].

Bacterial infection is one of the main factors that triggers inflammation and can stall this phase if not treated correctly [29]. Although some bacteria are part of the normal microbiota of the skin, when the epithelium is broken, they immediately invade the tissue, causing a symbiotic imbalance (bacteria-human). Microorganisms contaminate the wound bed and grow exponentially, with acute colonization that can later end in infection [30]. *S. aureus*, *P. aeruginosa*, *S. epidermidis*, and *E. coli* are organisms that have been found in infected wounds [31]. *S. aureus* and *E. coli* were sensitive to 3.49 mg/mL (5 μL) BSEO; however, there were minor significant differences with respect to chloramphenicol. Notably, only *E. coli* produced an MIC of 0.25 mg/mL and an MBC of 0.5 mg/mL. Canales et al. [3] demonstrated the inhibition of bacterial growth of these same strains when exposed to the essential oil of *B. morelensis*, with *E. coli* being one of the most sensitive bacteria, giving an MIC of 0.125 mg/mL and an MBC of 0.25 mg/mL.

In the EOBS, nine different terpenes were identified, corresponding to 83.8% of the total, which differs from another study in which a more significant number of volatile compounds was identified in the essential oil and resin of this same species [32]. α-phellandrene was the main compound in this study, while β-phellandrene was found in the essential oils of the leaves and resins of *B. shclechtendalli* [10], and in the essential oil of the bark of *B. morelensis*, both isomers were present [3,29]. What appears to be that these monoterpenes are characteristic of the genus *Bursera*. Factors such as temperature, the age of the plant, the range of volatility, and the process to which they are subjected can present many differences within the chemical composition [32]. The chemical composition of essential oils is a mixture of monoterpenes (mainly), and they present one or two primary compounds that are generally those that determine their biomedical properties [2]. However, interactions with lower abundance components can influence their biological activities [3]. For this reason, in this work, a bibliographic search of the metabolites identified in BSEO was carried out to know the possible targets that they present within the three phases of healing; each of the main aspects was highlighted, inflammatory phase: a reduction in the overproduction of NO and ROS, suppression of proinflammatory cytokine activity, COX-2 pathway blockade, and antibacterial activity. Proliferative phase: the proliferation of fibroblasts and formation of capillaries, and in the remodeling phase: collagen production and tensile strength.

There are fewer studies that have been carried out in the laboratory that demonstrate that these mixtures truly have synergistic effects compared to the pure compounds due to their structural complexity, ability to form bonds and stereochemistry, since some of them are optically active or inactive in nature [4,33]. Although in this work no compound was isolated or pure compounds were mixed to determine a possible synergistic interaction, based on the consulted bibliography, the mechanisms of action that the compounds are probably having and the possible synergies between them are schematically suggested [33].

This research proposes that the mixture of α-pinene and α-phellandrene could block the COX-2 and LOX pathways, preventing the formation of prostaglandins and leukotrienes [12,13,31]. Similarly, α-phellandrene and limonene could reduce ROS and NO levels [13,14], as well as the combination of α- and β-pinene has been shown [4] to be effective against this target. With the same effect but independently, camphene [17] and sabinene [11] reduce these radicals.

Additionally, α- and β-pinene have been shown to have synergistic [4] antibacterial activity against methicillin-resistant *S. aureus* (MRSA), which occurred in a shorter time than that with the isolated compounds. It has also been reported that camphene exhibits antimicrobial activity and has even been proposed as an excellent molecule against biofilms of *C. albicans*, [34]. Additionally, borneol [17] and limonene [4] have shown antibiotic effects when isolated. It is speculated that essential oils act on bacteria by causing membrane rupture due to their lipophilic components, which occurs through three pathways: increasing membrane permeability to small ions, affecting membrane stability and interrupting the packing of the lipid bilayer [2]. Therefore, it is most likely that BSEO is acting in this way to produce its antibacterial effect.

In the proliferative phase, phellandrene, borneol, and camphor help the proliferation of fibroblasts [14,17,19] and limonene in the formation of blood capillaries [16]. In the same way, limonene, borneol, and camphor help create balance in the transition to the remodeling phase by stimulating collagen production and increasing tensile strength [16,17,19]. On the other hand, it is important to point out that limonene is the only monoterpene that has been shown to have biological activities within the three phases of the healing process, since it was reported in five out of eight biological activities, despite the fact that it is not the majority compound. However, it has been identified within the chemical composition of various essential oils and resins from different species of the genus *Bursera* [3,32,35]. Therefore, it is very likely that it is responsible for the healing effect on wounds and, therefore, it is used in traditional Mexican medicine.

In general, natural products are widely used for prolonged periods without considering the health problems that can lead to an uncontrolled dosing regimen. These products are currently not considered medicines but natural products with free access and, therefore, are the responsibility of the final consumer [36], which is presented in a greater way when the natural product is applied topically and is classified as innocuous. Therefore, it is essential to carry out dermal toxicological tests. Although hexane extracts of several *Bursera* species, including *B. schelenchtendalii*, have shown cytotoxicity in vitro studies [37], according to OECD protocol 402, BSEO was shown to be safe at the maximum applied dose (2000 mg/kg), as there were no changes in weight or animal welfare parameters. Essential oils from other plant species have been tested with the same protocol and maximum dose and have been reported to be safe without showing lethality [36,38]. Alpha-phellandrene [14], alpha-pinene [12], limonene [4], sabinene [11] and camphene [18] did not exhibit any cytotoxic effect against macrophages, keratinocytes, or fibroblasts [11]. These results are extremely important because the plant *B. schlechtendalii* is used in communities, and people apply the essential oil from its branches to wounds without discrimination.

## 4. Materials and Methods

### 4.1. Plant Material

The collection of plant material was carried out in the municipality of San Antonio Nanahuatipam, located in the Cañada Oaxaqueña region. Collection was carried out during the rainy season, so the trees had leaves and fruits. The youngest stems were packed in plastic bags and protected with plastic films for transport to the laboratory; they were kept in a cold room (4 °C) until the essential oil extraction process. A specimen of the plant was deposited and identified in the herbarium from Facultad de Estudios Superiores Iztacala, UNAM registration number 3226-IZTA.

### 4.2. Obtaining the Essential Oil by Hydrodistillation

The stems were cut off into pieces of approximately 2 cm. Then, they were placed inside a 1000 mL flat bottom balloon flask with enough deionized water to cover the material. The flask was adapted for double condensation. For condensation, the flask was connected to a recirculating cold-water fountain and placed on the warming blanket [39] (SEV-Prendo, MC301-9, Puebla, Mexico). The distillation process was carried out for 30–40 min at 60 °C. The *B. schlechtendalii* essential oil was collected in 5 mL vials and the aqueous phase was discarded. The essential oil yield was calculated using the following formula:

$$Extraction\;yield=\frac{a}{b}\;\left(100\right)$$

where *a* is the weight (g) of the essential oil obtained and *b* is the weight (g) of the raw material. The oil was kept at −4 °C protected from light.

A total of 2.5 kg of young branches were collected, and 12.989 g of *B. schlechtendalii* essential oil was obtained for a yield of 0.52%.

### 4.3. Experimental Animals

All animals were purchased from the animal husbandry laboratory of Facultad de Estudios Superiores Iztacala, UNAM, Mexico. The animals used for the acute dermal toxicity test were female Wistar rats weighing 200–220 g. For the ear edema and wound healing efficacy experiments, healthy male CD-1 mice were used. The animals were allowed to acclimatize for one week before each test. They were handled according to the guidelines of NOM-062-ZOO-1999 on the handling and care of animals on a light-dark cycle of 12 h at a constant temperature (22 ± 2 °C) and relative humidity (50 ± 5%). The standard diet of pellets and drinking water was provided ad libitum during all experiments. All guidelines were followed and approved by the Ethics and Research Committee of the Escuela Nacional de Ciencias Biológicas, IPN (CEI-ENCB) (registration number ZOO-004-2020) and the Ethics Committee of the Facultad de Estudios Superiores Iztacala, UNAM (CE/FESI/052019/1295).

### 4.4. Wound Healing Activity

#### 4.4.1. Measurement of Wound Contraction

A total of 18 male mice were randomized into three groups: the first received 10 µL of 50% *B. schlechtendalii* essential oil (BSEO-50), the second received 0.5 mg of dexpanthenol (Bayer, Mexico) (positive control), and the third received 10 µL of mineral oil (vehicle). The dorsal hair of the mice was shaved with an automatic shaver and immediately depilated with a depilatory cream 24 h before the test [40].

The animals were anesthetized with 5% isoflurane (PISA, Mexico City, Mexico) by inhalation. An approximately one-centimeter long cut was made with a scalpel on the dorsal part of the depilated area (initial wound size). The next day, topical application of the different treatments on the wounds began and were performed every 12 h for 14 days. Wound closure was monitored and photographed with Motic Images Plus 3.0 software and measured daily with ImageJ2 software (https://imagej.net/software/imagej2/, accessed on 7 June 2023) (specific day wound size). On Day 15, all mice were sacrificed in a CO_2_ chamber.

Wound contraction (%) was calculated by the following equation:

$$\%\;wound\;contraction=\frac{initial\;wound\;size-specific\;day\;wound\;size}{initial\;wound\;size}\times 100$$

##### Histological Analysis

Tissues were removed from the wound section, fixed in 10% formaldehyde, and processed for histological analysis to identify the architecture of the skin layers, edema, immune cell infiltration, and blood vessel formation. Wound closure length was measured with ImageJ2 software.

#### 4.4.2. Tensiometric Method

In this study, 24 mice were randomly divided into four groups with six animals each (*n* = 6) and treated topically with 10 μL of BSEO-50, 0.5 mg of dexpanthenol/Bayer, Mexico) (positive control), or 10 μL of mineral oil (vehicle), with one group of mice left unwounded. The conditions of animal preparation, wound type, experimental time and method of sacrifice were similar to those described previously.

After sacrifice, the tensile strength of the wounds was measured using the water flow technique. Then, the percentage of wound healing was calculated with the following equation:

$$\%\;wound\;healing\;efficacy=\frac{A0\times An}{A0}\times 100$$

where *A*0 refers to the initial wound size and *An* to the wound size on a specific day.

### 4.5. Assessment of TPA-Induced Inflammation in Mice

The inflammation test was performed with 12-O-tetradecanoylphorbol-13-acetate (TPA) (Sigma-Aldrich, Saint Louis, MO, USA), which increases skin thickness due to edema formation and inflammation [41]. Thirty mice were divided into five groups of six individuals each (*n* = 6) and fasted for 4 h before the test. The control group consisted of the left ears without any treatment. Ten microliters of TPA was topically applied to the internal and external parts of the right ear and left on for 30 min. Then, the following treatments were applied: BSEO-50 (50 μL), diclofenac (0.116 mg) (Voltaren, Mexico) as the reference drug, and mineral oil as the vehicle (50 μL). After 4 h of treatment, the mice were sacrificed by cervical dislocation, and samples were removed with a biopsy punch.

All samples were prepared for conventional histological analysis (H&E staining), and with ImageJ2 software, microscopic observations and measurements of inflammatory thickness were carried out. The anti-inflammatory effect was calculated from the TPA group (a), which was considered 100% inflammation, control group (b), and groups with different TPA treatments (c). Using the following formulas, the values below were calculated:

Edema A induced by TPA alone: (a − b);
Edema B induced by TPA plus treatment: (c − b);
Anti-inflammatory percentage (%) = [(Edema A − Edema B)/Edema A] ∗ 100.

### 4.6. Antibacterial Activity

#### 4.6.1. Microorganisms

The following strains of bacteria were used: *Staphylococcus aureus*, *Staphylococcus epidermidis* (both isolated from a clinical case), *Pseudomonas aeruginosa* (donated by the CINVESTAV), and *Escherichia coli* (isolated from a clinical case).

#### 4.6.2. Evaluation by the Kirby–Baüer Agar Diffusion Method [42]

The microorganisms were cultured in Müeller Hinton broth (Bioxon 260-1) (Becton Dickinson, Mexico City, México). Cultures were adjusted to a turbidity comparable to McFarland no. 0.5 standard (Sigma-Aldrich, Saint Louis, MO, USA) with sterile saline. Subsequently, Petri dishes containing 4 mm thick Müeller Hinton agar (Bioxon 260-1) were impregnated with the microbial suspensions. Immediately after, previously sterilized 5 mm diameter sensidiscs (Whatman No. 5 paper) (Sigma-Aldrich, Saint Louis, MO, USA) were placed on the agar, which were impregnated with 5 μL of BSEO. Chloramphenicol (Sigma-Aldrich, Saint Louis, MO, USA) -impregnated sensidiscs (25 μg) were used as a positive control. The plates were incubated at 37 °C for 24 h, and then the diameters of any resulting zones of inhibition (mm) were measured. Each experiment was performed in triplicate.

#### 4.6.3. Estimation of the Minimum Inhibitory Concentration (MIC) and Minimum Bactericidal Concentration (MBC)

The following concentrations of BSEO were prepared in vials with 1 mL of Müller-Hinton broth and dimethylsulfoxide (DMSO) (Sigma-Aldrich, Saint Louis, MO, USA) at a final concentration of 0.2% *v*/*v*: 10, 8, 6, 4, 2, 1, 0.5, and 0.25 mg/mL. Subsequently, 150 µL and 10 µL aliquots and 10 µL of inocula of the bacterial culture (1.5 × 105 CFU/mL) were taken and placed in 200 µL microcentrifuge tubes [43]. Three replicates of each concentration were carried out. The microcentrifuge tubes were incubated for 24 h at 37 °C. Subsequently, 40 µL of a 0.08% solution of oxidized tetrazolium salt (TTC) was added for 30 min of incubation at 37 °C to confirm cell viability. To determine the MIC and MBC, the procedure mentioned by Espinosa et al. (2022) was followed.

### 4.7. Essential Oil Analysis

BSEO-50 was quantitatively and qualitatively analyzed with an Agilent Technologies 6850 gas chromatograph (USA) equipped with an HP-5 ms column (Agilent Technologies, Santa Clara, CA, USA, 30 m × 0.25 mm i.d. and 0.25 µL film thickness; USA). The elution program was as follows: 20 °C for 3 min, increasing to 300 °C at a rate of 20 °C/min. The injector temperature was 280 °C. Peak area percentages were determined using RTE integrator software (https://www.agilent.com.cn/, accessed on 7 June 2023) (Agilent Technologies, Santa Clara, CA, USA). Compound identification was carried out by mass spectrometry with a 5975 C gas chromatograph (Agilent Technologies, Santa Clara, CA, USA). The samples were ionized by electronic impact at 70 eV at a temperature of 230 °C. An HP-5 ms column was used (Agilent Technologies, 30 m × 0.25 mm i.d., and 0.25 µL film thickness, Santa Clara, CA, USA). The separation conditions were as follows: initial temperature of 300 °C for 2 min, increasing at a rate of 20 °C/min to reach 230 °C and then at a rate of 8 °C/min to reach 280 °C, and this temperature was maintained for five minutes. Helium was used as the mobile phase at a flow rate of 1 mL/min, and a split-type injector was used. The volume of the injected sample was 0.5 mL. The identification of chemical components was performed by referencing the NIST library 8.0 database (Technology Standard National Institute) (Gaithersburg, MD, USA) and by comparisons with the retention times from the HP-5 ms column.

### 4.8. Proposals and Descriptions of the Mechanisms of Action of BSEO in the Healing Process

According to the results of the healing efficacy and anti-inflammatory activity evaluations and the chemical components of BSEO-50, a bibliographic search of each compound with possible antimicrobial, antioxidant, or anti-inflammatory activity or cell migration effects was carried out. Subsequently, proposals of the mechanisms of action during each phase of the healing process were described.

### 4.9. Acute Dermal Toxicity

The OECD 402 protocol was followed for the toxicity test [44]. Nine animals were used and randomly distributed into three groups with 3 rats each with animal per cage. All animals had their backs shaved with an automatic razor 24 h before the experiment. The maximum BSEO-50 dose was applied in the BSEO group (2000 mg/kg), the vehicle group received mineral oil (500 mg/kg), and the control group did not receive treatment. Each dose was applied evenly to the shaved dorsal area, covering only 10% of it, using a cotton dressing. The dressing remained in place for one hour after exposure to ensure essential oil absorption.

Consequently, the animals were monitored for 14 days and weighed three times a week. The animals were observed for mortality and any toxic or deleterious effects with special attention given to the first 4 h and then once daily for a period of 14 days following topical application. Treatment-induced sequelae were monitored for irritation, edema, redness, fistulas, erythema, burns, etc. At 15 days, the animals were sacrificed by cervical dislocation.

### 4.10. Statistical Analysis

The results of the inflammation assay and healing efficacy test are expressed as the mean ± S.D.M. Data analysis was performed using the Wilcoxon *t* test and Student’s *t* test, respectively, and a value of *p* < 0.05 was considered to indicate statistical significance. All analyses were carried out using GraphPad Prism 7 software.

## 5. Conclusions

- BSEO-50 (*Bursera schlechtendalii* essential oil at 50% *v*/*v*) demonstrated a significant healing effect in the murine model, as evidenced by improved wound closure length, increased tensile strength, and antibacterial activity against clinically relevant strains.
- Phytochemical analysis revealed the presence of terpene compounds in BSEO-50, which were evaluated together, and the effects observed can be attributed to a synergistic relationship between them.
- Several terpenes present in BSEO-50, including limonene, borneol, and camphene, demonstrated antibacterial activity.
- BSEO-50 increased the tensile strength of the scar, and camphene probably is responsible since it has been demonstrated to promote collagen production.
- Of the compounds identified in the BSEO-50, there are reports that during the proliferative phase of wound healing, α-phellandrene and camphor were found to stimulate fibroblast proliferation, while camphor also contributed to the production of new capillaries.
- The experimental results obtained, combined with the existing literature on each identified compound in BSEO-50, support the ability of *Bursera schlechtendalii* to effectively heal superficial wounds.

## Figures and Tables

**Table 1:** Antibacterial activity of BSEO.

	Mean ± Standard Deviation			
Bacteria	Positive Control Chloramphenicol 5 µL	Inhibition Halos (mm)	MIC (mg/mL)	MBC (mg/mL)
*S. aureus*	20.4 ± 0.5	7.3 ± 0.5 ^a^	>10	>10
*S. epidermidis*	21.8 ± 0.4	NA	ND	ND
*P. aeruginosa*	13.2 ± 0.4	NA	ND	ND
*E. coli*	20.8 ± 0.4	11.6 ± 0.5 ^a^	0.25	0.5

Effect of the essential oil of *B. schlechtendalii* on bacterial strains. ^a^ There are significant differences with respect to the control group (*p* < 0.05). NA: no activity. ND: not determined, MIC: minimum inhibitory concentration, and MBC: minimum bactericidal concentration. (>) A higher concentration is needed to determine the MIC and MBC.

**Table 2:** Identified compounds from *B. schelechtendalii* essential oil by GC-MS.

Name	Retention Time (min)	Family	Molecule	% Total
Sabinene	4.740	Monoterpene	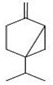	5.276
α-Pinene	4.868	Monoterpene	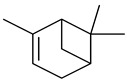	5.128
Camphene	5.093	Monoterpene bicyclic	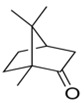	3.797
β-Pinene	5.413	Monoterpene	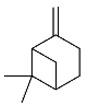	11.620
α-Phellandrene	5.694, 5.782	Monoterpene	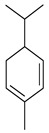	31.181
Limonene	5.926	Terpene	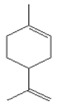	7.155
p-Menthane	5.974	Terpene	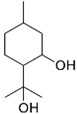	16.471
Camphor	7.161	Terpene	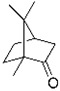	2.201
Borneol	8.219	Terpene	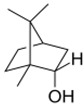	0.962
Total				83.791

**Table 3:** Reported biological activities of the chemical compounds found in the BSEO of each phase of the wound healing process.

	Inflammatory Phase				Proliferative Phase		Remodeling Phase		References
Chemical Compound	Reduction NO and ROS	Decreased Inflammatory Interleukins	Suppression in COX/LOX Pathway	Anti-Bacterial Activity	Fibroblast Proliferation	New Capillary Vessels	Collagen Production	Increased Tensile Strength	
Sabinene	X	X							[11]
α-Pinene		X	X						[12]
β-Pinene	X (with α-pinene)			X (with α-pinene)					[4,13]
α-Phellandrene	X (with limonene)	X	X (with α-pinene)		X				[12,13,14,15]
Limonene	X	X		X		X	X		[4,15,16,17]
Borneol				X	X		X	X	[17]
Camphene	X			X					[18]
Camphor					X		X	X	[19]

**Table 4:** Observations of changes in animal welfare parameters in female rats treated with 2000 mg/Kg of BSEO.

Parameters	30 min			2 h			4 h			24 h			48 h			72 h			7 Days			14 Days		
	C	V	O	C	V	O	C	V	O	C	V	O	C	V	O	C	V	O	C	V	O	C	V	O
Skin irritation	N	N	2 × 1 n	N	N	1 × 2 n	N	N	N	N	N	N	N	N	N	N	N	N	N	N	N	N	N	N
Piloerection	N	N	2 × 1 n	N	N	2 × 1 n	N	N	1 × 2 n	N	N	N	N	N	N	N	N	N	N	N	N	N	N	N
Eyes	N	N	N	N	N	N	N	N	N	N	N	N	N	N	N	N	N	N	N	N	N	N	N	N
Mucosal	N	N	N	N	N	N	N	N	N	N	N	N	N	N	N	N	N	N	N	N	N	N	N	N
Irritation	N	N	N	N	N	N	N	N	N	N	N	N	N	N	N	N	N	N	N	N	N	N	N	N
Breathing	N	N	N	N	N	N	N	N	N	N	N	N	N	N	N	N	N	N	N	N	N	N	N	N
Tremors	N	N	N	N	N	N	N	N	N	N	N	N	N	N	N	N	N	N	N	N	N	N	N	N
Seizures	N	N	N	N	N	N	N	N	N	N	N	N	N	N	N	N	N	N	N	N	N	N	N	N
Salivation	N	N	N	N	N	N	N	N	N	N	N	N	N	N	N	N	N	N	N	N	N	N	N	N
Diarrhea	N	N	N	N	N	N	N	N	N	N	N	N	N	N	N	N	N	N	N	N	N	N	N	N
Lethargy	N	N	1 × 2 n	N	N	N	N	N	N	N	N	N	N	N	N	N	N	N	N	N	N	N	N	N
Somnolence	N	N	N	N	N	N	N	N	2 × 1 n	N	N	N	N	N	N	N	N	N	N	N	N	N	N	N
Coma	N	N	N	N	N	N	N	N	N	N	N	N	N	N	N	N	N	N	N	N	N	N	N	N
Mortality	N	N	N	N	N	N	N	N	N	N	N	N	N	N	N	N	N	N	N	N	N	N	N	N

C = control, V = vehicle, O = BSEO, N = normal, 1 × 2 = one affected animal two normal animals, and 2 × 1 = two affected animals one normal animal.

## Data Availability

The data used to support the findings of this study are available from the corresponding author upon request.
